# From imitation to meaning: circuit plasticity and the acquisition of a conventionalized semantics

**DOI:** 10.3389/fnhum.2014.00605

**Published:** 2014-08-08

**Authors:** Ricardo R. García, Francisco Zamorano, Francisco Aboitiz

**Affiliations:** ^1^Centro de Estudios Cognitivos, Facultad de Filosofía y Humanidades, Universidad de ChileSantiago, Chile; ^2^División de Neurociencia, Centro de Investigación en Complejidad Social, Facultad de Gobierno, Universidad del DesarrolloSantiago, Chile; ^3^Departamento de Psiquiatría, Escuela de Medicina, y Centro Interdisciplinario de Neurociencia, Pontificia Universidad Católica de ChileSantiago, Chile

**Keywords:** imitation, language, circuit plasticity, onomatopoeia, pantomime, semantics

## Abstract

The capacity for language is arguably the most remarkable innovation of the human brain. A relatively recent interpretation prescribes that part of the language-related circuits were co-opted from circuitry involved in hand control—the mirror neuron system (MNS), involved both in the perception and in the execution of voluntary grasping actions. A less radical view is that in early humans, communication was opportunistic and multimodal, using signs, vocalizations or whatever means available to transmit social information. However, one point that is not yet clear under either perspective is how learned communication acquired a semantic property thereby allowing us to name objects and eventually describe our surrounding environment. Here we suggest a scenario involving both manual gestures and learned vocalizations that led to the development of a primitive form of conventionalized reference. This proposal is based on comparative evidence gathered from other species and on neurolinguistic evidence in humans, which points to a crucial role for vocal learning in the early development of language. Firstly, the capacity to direct the attention of others to a common object may have been crucial for developing a consensual referential system. Pointing, which is a ritualized grasping gesture, may have been crucial to this end. Vocalizations also served to generate joint attention among conversants, especially when combined with gaze direction. Another contributing element was the development of pantomimic actions resembling events or animals. In conjunction with this mimicry, the development of plastic neural circuits that support complex, learned vocalizations was probably a significant factor in the evolution of conventionalized semantics in our species. Thus, vocal imitations of sounds, as in onomatopoeias (words whose sound resembles their meaning), are possibly supported by mirror system circuits, and may have been relevant in the acquisition of early meanings.

## Introduction

In the last decade the evolution of human language has been a topic of increasing interest. This has focused on the evolutionary and neurocognitive foundations of human communication, and a wealth of comparative studies involving human and primate brains has intended to find a phylogenetic continuity between the structural networks subserving human language and neural circuits present in the primate brain. Other lines of research that consider other species of mammals, especially songbirds, have contributed to enlarge this complex theoretical framework. As a consequence, the comparison between humans, non-human primates, vocal learning birds and other species has favored the emergence of several theories, some involving the motor systems and others invoking cognitive processes. However, all of them have addressed auditory-vocal integration as a critical element for human language acquisition (Petkov and Jarvis, 2012).

In this paper, we discuss those aspects associated with the origin of a primitive form of learned semantics in the human lineage, understood as a rudimentary conventionalized system of symbols representing objects or events in the world. This is different from the innate referential vocalizations of some vocal non-learning primates, in which calls may signal the presence of specific predators (Seyfarth and Cheney, 2003a,b; see below). For this purpose, we propose the consideration of three major issues in order to place our discussion in an evolutionary context: first, a general approach to different theories seeking to explain the similarities and differences of vocal learning in a broad range of species including humans, non-human primates and other animals. Thus, we place the emergence of conventionalized semantics in a phylogenetic framework encompassing both behavioral and neurobiological foundations. In our view, vocal learning is a critical point in the origin of spoken language and meaning. Second, we discuss the structural homologies between the human brain networks associated to language and the premotor and temporo-parietal connections that are present in the primate brain. Two lines of evidence can be identified in this domain of research, making emphasis on different aspects with regard to the critical elements in the acquisition of language: one underlines the emergence of auditory-premotor circuits in the macaque brain as a pivotal step in language origins (Aboitiz and García, 1997; Aboitiz et al., 2006), and another claims that human language evolution is rooted in the development of the hand and gesture motor system (Arbib, 2005, 2011). In a third section, we will extend this conceptual framework by including a discussion about the likely processes leading to the emergence of primitive meaning in human communication. Here, we will consider putative contributing factors like pantomimes and onomatopoeias, neural plasticity associated to vocal learning, the social control of attentional resources and finally the development of a plastic phonological sensorimotor circuit featuring a strong auditory working memory capacity as a critical factor supporting the establishment of an increasingly complex referential semantic framework.

## Vocal learning species

Vocal learning is a key topic for the evolution of human language. This makes reference to the ability to acquire vocalizations through imitation rather than by instinct (Jarvis, 2004). This skill is found in some species of mammals (humans, bats, and cetaceans) and birds (parrots, hummingbirds and songbirds). Petkov and Jarvis (2012) recently reviewed motor and other neurobiological theories previously proposed for language evolution. In their review, the authors distinguished between vocal learning and auditory learning, and described the distribution of these traits among different species. They argue that auditory learning is widespread in higher vertebrates, while vocal learning capacity is restricted to some lineages. Furthermore, vocal learning is not an all-or-none ability, as there are varying degrees of vocal learning capacity in different species.

Considering that mammalian and avian vocal learning species are distantly related, it has been proposed that vocal learning evolved independently from vocal non-learner ancestors, either in the three vocal learning groups of mammals or in the taxa of the three aforementioned vocal-learning birds. The foundations for this hypothesis come from avian neuroanatomical evidence specifying a dedicated vocal-learning circuit specific for songbirds. In fact, Jarvis (2004) claims that the three groups of vocal learning birds have seven similar, but not identical, vocal cerebral nuclei distributed within two vocal pathways: one anterior and the other posterior. While the anterior vocal nuclei are part of an anterior forebrain pathway loop connecting pallial, striatal and thalamic regions and participate in song learning and sequencing, the posterior nuclei are connected to vocal motor neurons of the brainstem and control song production (see Jarvis, 2004 for a detailed description). In the posterior vocal pathway, there is a projection from the robust nucleus of the arcopallium (RA) to motor neurons in the XII nerve nucleus that control the muscles of the syrinx. Interestingly, the vocal learning pathways described above have not been found in vocal non-learning birds such as chickens and pigeons (Jarvis, 2004). Finally, Jarvis (2004) identifies an auditory pathway that is highly conserved among songbirds and other bird species.

In humans, a similar subdivision of anterior/posterior vocal pathways was proposed by Jarvis (2004) with an anterior vocal pathway, which connects the premotor cortex (including Broca’s area) and surrounding regions with the anterior basal ganglia and anterior thalamus; and a posterior vocal pathway that extends from the face motor cortex to the brainstem. This latter pathway sends direct projections from the face area in BA 4 (from a region called laryngeal motor cortex, LMC), to the nucleus ambiguus in the brainstem. The LMC is linked to the production of vocalizations when stimulated (Simonyan and Horwitz, 2011). Thus, the posterior vocal pathway takes control of speech, whereas the anterior pathway is proposed to participate in speech learning.

It is interesting to note that recent research has revealed that adult male mice possess some basic skills which allow them to modify and maintain the spectral contents of their ultrasonic vocalizations (Arriaga and Jarvis, 2013). Furthermore, mouse ultrasonic vocalizations are represented in cortical regions including the motor cortex (perhaps analogous to the LMC in humans) and in striatal regions, and there is a projection from vocal motor cortex to the brainstem vocal motor nucleus ambiguus (Arriaga and Jarvis, 2013). Interestingly, the insertion of a human variant of the language-related FoxP2 gene in mice results in shifts and modulation of pup ultrasonic vocalizations and in local architectural changes in the striatum (Fischer and Hammerschmidt, 2011).

No homolog of the LMC has been yet described in non-human primates, although further research is needed to confirm this. Based on these findings, some researchers have claimed that the evolution of spoken language in humans is associated with the development of a direct projection from LMC to nucleus ambiguus (Jarvis, 2004; Simonyan and Horwitz, 2011). In support of this sort of evidence, some motor theories about the origin of vocal learning have been recently proposed, which will be discussed in the next section.

## Motor theories about vocal learning

A theory about vocal learning across species has been proposed by Feenders et al. (2008), who describe a general motor system in both vocal-learning and non-vocal learning birds that is located adjacent to the vocal motor pathway of vocal learners. These areas display expression of some immediate early genes (IEG) with body movements, while the same genes become expressed in vocal learning nuclei of songbirds when they sing (Jarvis et al., 2000). Furthermore, in songbirds, these body-movement associated areas appear to be organized in anterior and posterior pathways, in paralell with the adjacent vocal motor nuclei. Based on these findings, Feenders et al. (2008) propose that brain systems dedicated to vocal learning in distantly-related bird species evolved as specializations of preexisting motor systems inherited from a common ancestor, and are involved in vocal movement control and probably in motor learning. Feenders et al.’s (2008) theory prescribes that the three lineages of vocal learning birds evolved independently similar cerebral systems, but these were derived from a somatic motor network inherited from a common ancestor. Moreover, they claim that this proposal may be extended to mammals, and in particular, to humans: the main vocal learners. Additional evidence has shown that in zebra finches, some vocal learning nuclei like HVC and RA activate both in song production and in a learned food aversion task, while other nuclei important for vocal plasticity like LMAN and Area X activate only during singing (Tokarev et al., 2011). The authors claim that these findings indicate that some vocal control nuclei participate in non-vocal learning, thus existing some overlap between vocal learning and non-vocal learning nuclei. Furthermore, this is consistent with the notion that parts of the brain circuitry for song learning originated from networks related to feeding. With regards to anatomy, these suggestions agree with our original interpretation that part of the language-related Broca’s region and its homolog in other primates (area 44), derive from the ventral premotor cortex (Aboitiz and García, 1997). From a behavioral perspective, Feenders et al. (2008) likened their proposal to the gestural theory for the origin of spoken language alongside the mirror neuron hypothesis, to argue that gestural behavior in humans and non-human primates is a precursor for the acquisition of speech and language (Arbib, 2005, 2011; Gentilucci and Corballis, 2006).

## Connectivity of the human language areas

In the human, Broca’s area is located in the inferior frontal gyrus (IFG) and includes the pars opercularis (most posterior region), the pars triangularis (anterior) and the pars orbitalis (ventral). These subdivisions include Brodmann’s areas 44, 45 and 47, which fit the definition of the macaque ventrolateral prefrontal cortex (VLPFC). In the auditory region of the posterior temporal lobe, auditory area Tpt in the superior temporal gyrus (STG) has been associated with Wernickes area by some authors. This area is conceived as a multimodal cortical region receiving afferents from somatosensory and auditory regions (Galaburda and Sanides, 1980; Preuss and Goldman-Rakic, 1991).

**Figure 1:**
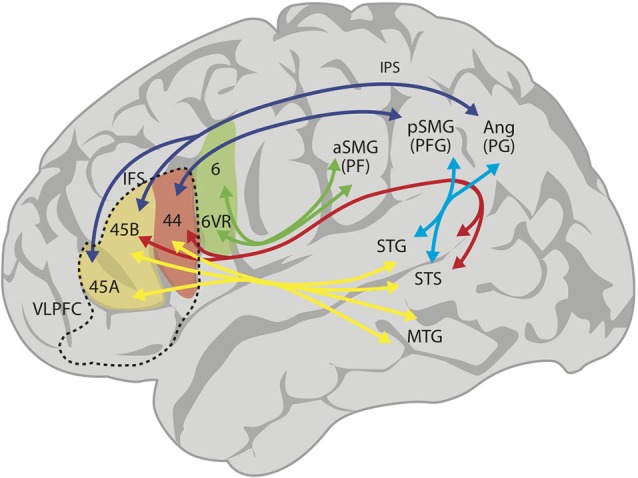
**Proposed schematic connectivity of the language-related regions in the human brain, based on Kelly et al. (2010)**. The superior longitudinal fasciculus (SLF) connects inferior parietal area PF (anterior supramarginal gyrus, aSMG) with premotor area 6v (green arrows), area PFG (posterior supramarginal gyrus) with area 44 and area PG (angular gyrus) with areas 45B and 45A (dark blue arrows). The arcuate fasciculus (AF; red arrows) connects the posterior superior temporal sulcus (STS) and gyrus (STG) with areas 44 and 45B. The middle longitudinal fasciculus connects STS and STG with PFG and PG (light blue arrows). Finally, there is a ventral projection via the extreme capsule (yellow arrows), connecting more anterior aspects of the STG, STS and middle temporal gyrus (MTG) with Broca’s region (areas 44 and 45). In summary, connecting the anterior and posterior language areas, there is a dorsal pathway with (i) a direct component (AF, red arrow); (ii) an indirect component (middle longitudinal fasciculus and SLF, light blue and dark blue arrows); and (iii) a multimodal ventral pathway (yellow arrows). The ventrolateral prefrontal cortex (VLPFC) is the area inside the broken lines, and includes areas 44, 45A and 45B, and area 47 (not colored). 6VR, area 6 ventral-rostral.

Over the last few years, the use of MRI tractography has been fundamental in describing the structural connectivity of the language circuits in the human brain (Catani and ffytche, 2005; Parker et al., 2005; Friederici et al., 2006; Anwander et al., 2007; Frey et al., 2008; Glasser and Rilling, 2008; Friederici, 2009). Consistent with other studies, Frey et al. (2008) described an arcuate fasciculus (AF) that connects the posterior STG (Wernicke’s region) to area 44 (posterior Broca’s region; Figure 1). However, these authors have also emphasized a robust projection from the inferior parietal lobe (IPL) and anterior temporal lobe to the VLPFC: there is a large projection from area PFG (anterior area 39, posterior supramarginal gyrus) in the IPL, via the superior longitudinal fasciculus (SLF) to area 44, and another from area PG (posterior area 39, anterior angular gyrus) to area 45 (this is subdivided into areas 45A and 45B; see Figure 1). Noteworthy to point out is that the IPL receives connections from temporal lobe auditory areas through the middle and inferior longitudinal fasciculi, thereby closing a circuit to area 44 (see Figure 1). These two projections, a direct one via the AF and an indirect one via the middle longitudinal fasciculus and the SLF to the VLPFC, make up the dorsal pathway for audition and language. In addition, there is a ventral pathway from anterior temporal areas that courses through the external capsule and ends in areas 47 and 45 (Figure 1). The dorsal auditory pathway has been considered a participant in phonological working memory, verbal articulatory processes and complex syntactic processing, while the ventral pathway is thought to be involved in speech recognition, verbal retrieval and simple grammatical processing (Buchsbaum et al., 2005a,b; Hickok and Poeppel, 2007; Saur et al., 2008).

Yet, the tractographic approach cannot precisely determine the specific areas of origin for the axonal connections in lateral temporal and inferior parietal cortices (Margulies and Petrides, 2013). Considering this, these authors implemented a resting-state functional connectivity analysis with the aim of unveiling the functional pattern of parieto-temporal-frontal connectivity. Their findings reveal that areas 45 and 44 display a distinct and unique profile, with area 45 functionally connected to the superior temporal sulcus (STS), the STG and middle temporal gyrus. In the inferior parietal cortex, area 45 was uniquely correlated with the angular gyrus (area PG in Figure 1), whereas area 44 was correlated with the supramarginal gyrus (area PFG in Figure 1). Interestingly, the ventral part of the precentral gyrus (area 6VR, see Figure 1), where the orofacial musculature is represented, is functionally linked to the rostral part of the supramarginal gyrus (area PF in Figure 1), while the primary motor cortex connects primarily with the postcentral gyrus (somatosensory cortex). Therefore, area 6VR is functionally linked with the somatosensory cortex on the post central gyrus with no direct communication with Broca’s area except indirectly through the premotor cortex. These results highlight the distinct patterns of connectivity in the two areas comprising Broca’s region, area 45 and 44, and predict functional differences between these regions. In fact, functional neuroimaging studies suggest an involvement of Broca’s region in the control of verbal fluency, with area 44 playing an important role in phonological fluency (Heim et al., 2008) and area 45 more involved in the control of retrieval of information from memory (Kostopoulos and Petrides, 2003).

## Homologs to human language circuits in the monkey

One of the most noticeable neuroanatomical findings in recent years is that brain regions, and circuits comparable to that of human vocal language-dedicated ones, have been confirmed in the monkey brain. These studies have revealed that the VLPFC of the macaque brain is structurally and functionally homologous to the IFG of the human brain (Romanski, 2012). In the macaque, the VLPFC occupies the inferior convexity of the prefrontal cortex and is subdivided similarly to the human frontal lobe: area 45, anterior to the inferior arcuate sulcus, area 12/47 just anterior to area 45 and ventral to area 46, and area 12 orbital in the most ventrolateral portion of the inferior convexity. Area 45 can be subdivided into areas 45A, extending rostrally in the adjacent inferior frontal convexity, and area 45B, lying caudally in the prearcuate bank (Petrides and Pandya, 2002; Petrides et al., 2005; Gerbella et al., 2010). These authors have also identified a dysgranular area 44 in the depth of the inferior arcuate sulcus, homologous to its homonym in the human.

Furthermore, recent evidence from neuroanatomical and imaging studies have contributed to clarify the understanding of temporo-parietal-frontal networks in primates. In the macaque, there is a double stream of auditory projections comparable to the organization of human language networks: a dorsal stream from auditory areas in the posterior superior temporal lobe that reaches dorsolateral frontal areas (8, 46) involved in eye movement control (Kaas and Hackett, 1999) and a ventral stream originating in anterior and middle areas of temporal lobe that sends visual and auditory inputs to areas 12 and 45 in the VLPFC (Romanski et al., 1999a,b). Interestingly, in areas 12 and 45 an auditory domain has been described in which neurons sensitive to vocalizations of conspecifics are intermingled with facial-sensitive neurons (O’Scalaidhe et al., 1997, 1999; Romanski and Goldman-Rakic, 2002; Romanski et al., 2005), suggesting an integration between vocalizations and orofacial gestures in the homolog of Broca’s area in humans (Sugihara et al., 2006). There is also a projection from caudal auditory cortex to the dorsal prefrontal cortex and even light projections from caudal auditory cortex to caudal area 45. In addition, the STS has direct projections to the VLPFC (Romanski et al., 1999a). However, such posterior temporal projections to the Broca’s area homolog have been considered to be weaker than in the human (see Aboitiz and García, 1997; Aboitiz, 2012).

Additionally, the IPL of the monkey has been shown to send a strong projection into the VLPFC. As in the human, the monkey IPL is subdivided into area PF, area PFG, area PG and finally, an area AIP in the intraparietal sulcus (Petrides and Pandya, 2009; see also Gerbella et al., 2011). Petrides and Pandya (2009) confirmed a projection originating in the inferior posterior parietal areas (PFG, PG) and arriving to areas 45 and 44 via the SLF. There is also a connection from the STS and posterior STG to the IPL that can potentially convey auditory information into the latter. As mentioned, connections from the ventral IPL and caudal STS running in the AF reach the VLPFC, but these are apparently much weaker in monkeys than in humans (Petrides and Pandya, 1999, 2002, 2009). In the ventral pathway, fibers via the extreme capsule and uncinate fasciculus that originate in the auditory and visual areas of the anterior and middle temporal lobes were found to end in areas 45, 47/12, and also in area 44 (Petrides and Pandya, 2009; see Figure 1). This is consistent with Webster et al.’s (1994) report that visual area TE in the anterior temporal lobe is connected with areas 8 and 45 in the inferior limb of the anterior bank of the arcuate sulcus and with area 12/47 in the inferior prefrontal convexity. Petrides and Pandya (2009) also suggested that the ventral projections to VLPFC are involved in memory retrieval, whereas the dorsal route (SLF and AF) suppports vocalization control only in humans.

Furthermore, using human resting-state technology, Neubert et al. (2014) report in macaque VLPFC regions a pattern of functional connectivity similar to areas in human ventrolateral frontal cortex largely associated with language. However, a noticeable species difference was found in how ventrolateral frontal areas coupled with posterior auditory association regions. Macaque auditory association areas in the superior temporal cortex correlated with regions in the anterior cingulate cortex (ACC), while human auditory association areas were strongly coupled with almost all ventrolateral frontal areas, confirming a human, species-specific enhanced auditory-motor vocal connectivity.

We must mention that overall, these findings in the human and in the macaque are anatomically consistent with, and confirm, our original hypothesis (Aboitiz and García, 1997), in which we claim a tripartite input into Broca’s region and its monkey homolog: one direct from the posterior superior temporal lobe via the AF, another one, an indirect route via the IPL and the SLF, and a ventral projection via the anterior temporal lobe. Furthermore, we claimed that the dorsal pathway had undergone an important alteration throughout the course of human evolution, particularly by increasing the relative size of the AF. As will be seen below, our hypothesis was that these innovations were fundamental for the development of a sensorimotor auditory-vocal circuit supporting phonological working memory, which was a key event in the acquisition of human language.

## The phonological loop, working memory and a primitive syntax

In a series of reports, we’ve claimed that the acquisition of a sensorimotor phonological loop was a key innovation in human language evolution (Aboitiz and García, 1997; Aboitiz et al., 2010). In line with trend-setting findings by Baddeley and collaborators (see Baddeley, 2003), we originally claimed that an expansion of auditory working memory capacity was of critical importance in learning and processing complex phonological sequences and a key step in the acquisition of speech. According to these claims, the development of a cortico-cortical auditory-vocal sensorimotor circuit was associated to the emergence of a functional phonological loop, which dramatically amplified the universe of possible vocalizations based on combinations of previously learned phenomena. Of note, this was also supported by the concomitant acquisition of voluntary control over the larynx and the supralaryngeal tract via a direct cortical projection to the brainstem vocal motor neurons.

In our view, the origin of this sensory motor circuit allowing for the rehearsal of newly learned phonological items in short-term memory, represents a cornerstone in human evolution because it made possible an inner speech skill that improved the elaboration of complex messages and the generation of new combinations of learned phonemes (Aboitiz, 2012). This circuit relies largely on the development of the dorsal pathway connecting Wernicke’s and Broca’s area, whereas the ventral pathway remains somewhat more conservative in evolution and, as in monkeys, was probably involved in vocalization processing and recognition in our ancestors (Romanski et al., 2005).

Consistent with this view, recent evidence has unveiled a limited capacity for auditory short-term memory in monkeys (Scott et al., 2012), which is in line with the concept that auditory working memory puts a limit to the complexity of vocal utterances. Nonetheless, although non-human primates are at best limited vocal learners (Hopkins et al., 2007; Snowdon, 2009; Petkov and Jarvis, 2012), research in auditory sequence learning capabilities has reported that non human primates are apparently capable of learning some simple artificial grammars. In fact, Wilson et al. (2013) have obtained evidence that Rhesus macaques can learn an auditory artificial grammar including branching relationships like those seen in the vocal production of songbirds (Hurford, 2012). We suggest that the increase in working memory capacity significantly amplified the ability to learn more complex sequences and to translate them into vocal motor patterns used in communication.

In this context, we have proposed that a phonological system provides a robust support for the emergence of an increasingly complex syntax based on distant dependencies between linguistic elements (Aboitiz et al., 2006; Aboitiz, 2012). From a neuroanatomical perspective, many imaging studies have shown Broca’s area involved in working memory processes linked to syntax. Recent evidence points to area 44 as a critical node for processing syntactic working memory, especially in the superior part (Friederici, 2004), while the dorsal pathway connected to it is involved in the syntactical processing of structures organized in a hierarchical manner (Friederici et al., 2006; Anwander et al., 2007).

Although the IPL may contribute to verbal working memory, it apparently holds a supporting role rather than that of storage system. In fact, any role for the IPL as a phonological storage mechanism has been recently challenged, as the only areas showing sustained activation during verbal working memory tasks are the STS and an area termed Spt in the STG, but not the IPL (Hickok and Poeppel, 2007; Hickok, 2009; see also Aboitiz et al., 2006, 2010). Accordingly, area Spt is thought to be an interface between the sensory and motor representations when the phonological ítems are on line, and may be part of area Tpt described above, perhaps even contributing fibers to the AF (Buchsbaum and D’Esposito, 2008; Buchsbaum et al., 2011).

## Mirror neurons, the hand-motor system and language

As mentioned previously, another line of research concerning language evolution has claimed the involvement of the motor system as a crucial step for human language development. This view has been strongly reinforced by the discovery of mirror neurons, a type of visuo-motor neuron associated with hand-grasping in monkeys. Mirror neurons were identified as being activated when an animal subject observed the experimenter or another animal making meaningful hand movements (di Pellegrino et al., 1992; Rizzolatti and Luppino, 2001; Rizzolatti and Craighero, 2004). These neurons are located in area F5 (BA 6v), a premotor area that is subdivided into regions Fa, Fb, Fc and Fd. Interestingly, Fa is adjacent to area 44, and has been conceived as an integration site for parietal sensory-motor signals with premotor and prefrontal information (Gerbella et al., 2011). Moreover, in the lateral aspect of Fa, face-selective mirror neurons have been detected whose activity increases when a monkey observes the communicative gestures of conspecifics (Ferrari et al., 2003; Rizzolatti and Craighero, 2004). Mirror neurons have also been detected in the rostral IPL where they are associated with both observation and execution of actions, and in the STS as a group of neurons responding to goal-directed hand movements (Perrett et al., 1990).

In humans, however, it has been difficult to search for mirror neurons for technical and ethical reasons. On the other hand, imaging and electroencephalographic tools have allowed for a visualization of the MNS related to observation of actions, imitation, and empathy (Rizzolatti and Craighero, 2004; Iacoboni and D’Apretto, 2006). The human MNS seems to be served by a wide network encompassing parietotemporal visual areas, the rostral IPL and inferior precentral and frontal gyri areas. Recently, a ventral pathway from the anterior temporal lobe has been suggested to support planning and decision making (Arbib, 2010) and the prediction of intentions and the goals of actions (Kilner, 2011). From a behavioral perspective, the MNS in humans is thought to be involved in the recognition of actions which is critical for decoding the other’s intention (Rizzolatti and Craighero, 2004).

On the basis of this conceptual framework, Rizzolatti and Arbib (1998) and Arbib (2005, 2011) have proposed that language emerged from neural circuits evolved from mirror neurons originally implicated in imitation and gestural behavior. In this sense, Arbib (2005, 2011) has proposed a progressive and sequential scenario starting from an imitation grasping system followed by a gestural system including pantomime as a key element leading to the development of a referential system. Finally, a “protosign” stage based on hand symbols would have somehow facilitated the emergence of vocal plasticity, configuring a “protospeech” stage that would evolve into modern speech (Arbib, 2005). Furthermore, Arbib claims that the MNS contains a neural mechanism for understanding actions and that this served as a blueprint for the origin of a simple syntax. To this respect, the use and manufacturing of tools may have had an important role in decomposing goal-directed actions in which the MNS participates. Tool use activates the inferior parietal and VLPFC and can be conceived of as a hierarchically-organized collection of body movements that might represent a rudimentary means of acquiring a nested and recursive syntactical structure (Stout and Chaminade, 2012).

Recently, Prather et al. (2008) observed a group of motor neurons in the swamp sparrow forebrain that fired along with the auditory note sequences in the sparrow’s repertoire, and on a similar note, the song sequences of other birds. These authors interpret these findings as evidence for mirror neurons, although more studies may be needed to confirm this possibility. Moreover, these neurons innervate striatal structures critical for song learning and their auditory-vocal properties seem to parallel those found in the MNS in the primate brain (Mooney, 2014). Furthermore, oral mirror neurons, that activate with facial gestures like lip smacking and feeding behavior, have been detected in F5 of the monkey, near area 44 (Rizzolatti and Craighero, 2004). This has suggested to some authors that neural control of communicative vocal behavior partly evolved from feeding-related circuits, and is consistent with the finding of food-associated activation of vocal learning nuclei in songbirds (Tokarev et al., 2011). Therefore, it is possible that the circuit associated with the phonological loop in humans contains mirror neuron-like elements that participate in generating an auditory-motor sensory interface (see also Aboitiz et al., 2006; Arbib, 2011; Aboitiz, 2012).

## A multi-modal communication system

As we have discussed up until this point, two lines of research have intended to account for the neurobiology of human language evolution: one that features an auditory-vocal mechanism as a pivotal step, and another based on hand symbols supported by neuro-mechanistic scaffolding provided by the MNS. However, it is our view that a more integrative perspective is necessary. In the current proposal, communication has evolved as a multi-modal, opportunistic process in both humans and monkeys, in which several possible mechanisms to convey socially relevant information are valid according to differing circumstances. In fact, functional and anatomical evidence indicates a confluence of facial and vocal information in the VLPFC (Sugihara et al., 2006) as well as the convergence of auditory, visual and somatosensory inputs in VLPFC (Romanski, 2012). More specifically, area 47/12 is a vocal-sensitive region with neurons responding to species-specific calls (Romanski and Goldman-Rakic, 2002; Romanski et al., 2005, reviewed in Romanski, 2007) and facial stimuli (O’Scalaidhe et al., 1997, 1999), whose activity has been confirmed more recently with fMRI (Tsao et al., 2008). Moreover, the body and hand representation in premotor area F5 of the monkey strongly suggests an integration of hand, face gestures and vocalization patterns (Aboitiz, 2012). Of interest in this context, a recent article reports that in the monkey, face-voice associations take place when the sender is a familiar individual but not for unfamiliar ones (Habbershon et al., 2013). Additional studies have shown that chimpanzees can match vocalizations with gesturing faces (Izumi and Kojima, 2004) and that the chimpanzee homolog of Broca’s area reaches a maximal activation during simultaneous gestural and vocal communicative actions, particularly when gestures and vocalizations are oriented toward calling the other’s attention (Taglialatela et al., 2008). In humans, area 44 has been found to be activated during mouth movements related to objects and in the imitation of gestures (di Pellegrino et al., 1992; Buccino et al., 2001). Another imaging evidence in humans has revealed that areas 44, 45 and 47 become activated when gestures and speech co-operate in communication (Willems et al., 2007; Gentilucci and Dalla Volta, 2008). Thus, in both humans and monkeys, a multimodal communication system makes use of overlapping neural circuits subserving both vocal and hand/body gestures (Aboitiz and García, 2009).

Finally in this section, recent studies have called attention to the voluntary control of the supralaryngeal tract in non-human primates, which is innervated by the hypoglossus and facial nuclei (Lameira et al., 2014). The supralaryngeal tract is required for the production of most consonants and may have contributed to learned vocal behavior long before the vocal folds in our ancestors. Furthermore, communicative lip smacking movements in monkeys are dissociated from throat movements and have a frequency close to five cycles-per-second, similar to lip movements during human speech and much more rapid than chewing (Ghazanfar et al., 2012; Morrill et al., 2012), which suggests a continuity between ancestral communicative facial gestures and modern human speech. Note again, that mirror neurons that activate with lip smacking have been described in the premotor cortex of monkeys (Rizzolatti and Craighero, 2004).

## Emergence of conventionalized semantics in human language evolution

Based on a multimodal perspective of communication, we will discuss the probable routes and mechanisms conducive to the capacity to utter learned, articulated sentences conveying meaning in a communicative context in human ancestors. This is a skill that characterizes our species but a rudimentary form of external reference can be found in other primates. In this section we will address evidence coming from both the hand/body gestures and the vocalization lines of research.

### Pointing behavior

Under the MNS paradigm/approach, gestures have been proposed to be critical for the origin of primitive meanings in humans. As Arbib (2011) claims, grasping activity and hand voluntary control play a fundamental role in motor actions demanding shared attention. This may have facilitated the development of pointing behavior as a derivation of hand-reaching, a simple behavior that allows making reference to the external world (Aboitiz, 2012). Pointing was possibly the impetus for other hand communicative gestures in an evolution from imitative behavior to simple, ritualized semantics (Aboitiz, 2012).

Pointing may be a non-communicative action when it incorporates only subject and object. Nonetheless, it becomes communicative in a three-way relationship including a subject who points, an object and an addressee (Cleret de Langavant et al., 2011). Fundamentally, pointing intends to share information about an object with another person, and in an evolutionary scenario it could represent a transition stage in the capacity of one to direct the other’s attention to a common object allowing an interchange of a particular meaning in a natural context. Interestingly, human infants and baboons share a right hand preference when they use pointing in a communicative task. In fact, the right hand preference was stronger for pointing tasks than for grasping objects, revealing left hemisphere dominance for communicative gestures (Meunier et al., 2012). Furthermore, communicative pointing seems widespread in non-human primates considering that pointing in the chimpanzee also conveys intentional and relational content (Leavens et al., 2004). Neural correlates of communicative pointing have implicated the right STS area at the temporoparietal junction (TPJ) in the IPL and right pre-supplementary motor area (pre-SMA), suggesting that pointing, as a communicative behavior, is involved in processes related to taking the other person’s perspective (Cleret de Langavant et al., 2011). These findings have been supported by imaging and electroencephalography techniques in a task binding gaze, gestures and emotions. In this study, directional cues like gaze and pointing activated the right parietal and pre-SMA, showing that the dorsal pathway is involved (Conty et al., 2012). In sum, pointing may represent a primitive stage in the development of learned semantics present in some non-human primates and infants. Fundamentally, it allows conveying information about objects incorporating an addressee in shared attention and social interaction.

### Pantomimes

A second aspect involved in the appearance of primitive semantics in language evolution regards pantomimic actions related to events and objects (Arbib, 2005). Pantomimes are gestures resembling the actions they represent, and evidence has revealed that in non-human primates these particular gestures are merely representations lacking abstraction, whereas in humans they involve abstract content and are related to a form of symbolic communication (Cartmill et al., 2012). Fundamentally, pantomimes are representational gestures and these kinds of motor actions are restricted to humans. In fact, primate gestures lack the representational nature of humans, although their gestures are used flexibly and intentionally (Cartmill et al., 2012). Among the types of human gestures—deictic like pointing, conventional and representational—the latter are critical for human communication and pantomimes are thought to represent a stage in the progression from manual action to meaningful spoken language (Cartmill et al., 2012). In this sense, the MNS hypothesis has been proposed to provide a neural basis for this transition (Arbib, 2005). Interestingly, using functional neuroimaging, Emmorey et al. (2010) reported that deaf signers displayed different patterns of brain activation when passively viewing pantomimes and ASL signs compared to hearing non-signers. Pantomimes strongly activated frontoparietal regions (MNS) in hearing non-signers, but only bilateral middle temporal regions in deaf signers. Presumably, life-long experience with hand/arm signs reduces or eliminates neural involvement of the MNS (Emmorey et al., 2010). Nonetheless, pantomiming, as a critical stage in language evolution, has been criticized because of evidence coming from chimpanzees. Experiments comparing children aged 2–4 years and chimpanzees in gesture imitation tasks revealed a restricted ability for chimpanzees in this type of imitative learning (Tomasello, 1996; Whiten et al., 1996). In our view, the particular relevance of pantomimes in the transition from gestural to vocal communication remains unclear. Probably, gestural pantomimes could be accompanied by the use of sounds making reference to the objects, opening, in this way, a stage where gestures and vocal activity co-occured. This could be relevant in the development of meaning in vocal behavior (Taglialatela et al., 2011; Aboitiz, 2012). Above, we have mentioned that Broca’s region activates strongly when subjects use speech and hand gestures concomitantly (Willems et al., 2007; Gentilucci and Dalla Volta, 2008). Furthermore, using functional MRI, Xu et al. (2009) have reported that pantomimes and spoken stimuli activated the same left lateralized network of inferior frontal and posterior temporal cortex suggesting that this perisylvian network represents a modality independent of semiotic system that plays a broader role in human communication.

### Vocalizations and onomatopoeias

From our perspective, vocalizations are a critical element in the acquisition of human language and meaning. Vocalizations could have enriched joint attention with others, especially combined with gaze direction. Related to this, the anterior cingulate cortex (ACC), a region involved in affect-related vocalizations in humans and monkeys (Yukie and Shibata, 2009), participates in the detection of incongruent stimuli or events that are contrary to expectations (Allman et al., 2001). Recall the aformentioned findings of Neubert et al. (2014), who found a strong, functional coupling between the VLPFC and the ACC in monkeys (and in humans). Hence, vocal behavior could make reference to socially salient situations or events that contradict predictions. In line with this, (Seyfarth and Cheney, 2003a,b) have found that vocalizations produced by vervet monkeys and baboons are not only emotional, but also referential, as the listener may extract external information from the calls, such as the presence of specific predators. However, as these authors assert, these vocalizations differ from human language in at least one aspect: the listener can acquire information from vocalizations, but the caller may not intend to provide it.

One step further, the capacity to produce onomatopoeia-like vocal imitations of sounds could have participated in the acquisition of early meanings in attentionally-demanding contexts (Assaneo et al., 2011). Exposure to onomatopoeias activate the left anterior STG, and bilaterally, the STS, the middle temporal gyrus and the IFG, areas implicated in the processing of verbal and non-verbal sounds (Hashimoto et al., 2006). It is tempting to propose that onomatopoeias may be supported by mirror neuron circuits on the basis of alleged temporal and frontal networks involved in the MNS of monkeys and, probably, humans as well (Arbib, 2005).

## Discussion

The evolution of human language and its underlying cerebral networks has been a matter of intense debate and discussion over the last few years. Although one approach has emphasized a predominantly “gestural” origin for language, and a second one has focused on the development of an auditory-vocal mechanism leading to human language, we, however, have indicated that an alternative perspective exists. We postulate a multimodal and opportunistic system of communication using manual signs and vocalizations in natural contexts, which could be a more plausible model for explaining human language evolution (Aboitiz, 2012). In this proposal, both gestural and vocal information coincide in the emergence of conventionalized semantics, leading to object-naming and eventually to describing the environment surrounding us. In our view, a fundamental event in semantics acquisition has been the development of plastic neural circuits subserving both gestural and auditory-vocal networks allowing complex human communication. In this frame, gestural-based actions like pointing and pantomimes cooperate dynamically with learned vocalizations. Eventually, the latter became of critical importance during human evolution, reaching a predominant role. Moreover, recent evidence has revealed that human vocal activity has considerable functional flexibility allowing human infants to control affective expression through early vocalizations (protophones) (Oller et al., 2013). These data strongly suggest that this functional flexibility appearing early in the first year of human life could be critical for the development of vocal language. Until now, such flexible affective expression of vocalizations has not been reported for any non-human primates. Furthermore, although both gestural and vocal communication were important in the establishment of a learned referential semantics, we argue that the advent of vocal learning, and more importantly, the expansion of verbal working memory capacity, were crucial events in the amplification of communicative signals into modern language.

Finally, and to differ from MNS exponents, we consider less likely the possibility that vocal plasticity appeared directly to support transmission of novel meanings in the context of an “open-ended” gesture-based communication system (termed the “proto-sign” stage), as Arbib (2011) and others have proposed. This possibility would imply that a very complex vocal system became recruited at once and out of nearly nothing, developing plastic and combinatorial capacity, while at the same time involving a semantic component. We prefer the alternative that this was achieved gradually whereby vocal learning coevolved with gestural communication, as it happens in other animals (Lipkind et al., 2013). In early humans, vocal learning capacity was possibly acquired in the context of mother-child bonding, individual recognition, and some other social requirements. Subsequently, through imitation-based onomatopoeias combined with gestural pantomimes, these vocalizations began to assimilate some type of primitive meaning. Importantly, superior vocal tract sounds associated with facial gestures, like lip smacking and others, may have been present from very early stages of language evolution and are likely continuous with some lingual or facial movements used in modern speech (Lameira et al., 2014). In our view, the gesture-based “proto-sign” stage specified by Arbib (2011) as a sequential link between pantomimes first and proto-speech last, is largely hypothetical and apparently not well defined in terms of its specific structure or examples. Furthermore, we have found no evidence that in primitive humans, gestural communication went much beyond what is observed in typical, modern speech-based human communication, neither in child development nor in the adult.

Thus, we concur with exponents of the MNS in acknowledging an important role of gestures and pantomimes in the origin of linguistic meaning, but consider that this is only part of the full story in which learned vocalizations worked together with gestures and significantly contributed to transmit meaning, both by inducing shared attention and by imitating sounds of physical objects. In other words, while the MNS hypothesis emphatically prescribes a sequential process, first via signs and then vocalizations, we prefer a scenario in which gestures and vocalizations coevolved from very early stages, with vocalizations leaving gestures behind concomitant with the development of a robust, functional phonological loop supporting verbal working memory. From then on, complex vocal messages and a primitive syntax began to emerge, rapidly leading to modern human language.

## Conflict of interest statement

The authors declare that the research was conducted in the absence of any commercial or financial relationships that could be construed as a potential conflict of interest.
